# Letter to the editor, Odell 2019

**DOI:** 10.1186/s12998-019-0271-x

**Published:** 2019-08-20

**Authors:** Peter Tuchin

**Affiliations:** Macquarie University, North Ryde, Sydney, Australia

Odell et al., should be commended on an important study for chiropractic treatment of chronic migraine [1]. However, in my opinion, there are some significant limitations in the design of the trial.

Firstly, with the trial having no placebo/sham group, the strength of the trial is decreased, and any potential conclusions will be significantly limited [2]. I suggest the authors consider adding a possible placebo/sham arm of the trial, which could include:

- Sham SMT [3]
- Detuned interferential/ultrasound [4]
- Placebo pharmaceutical, such as a “muscle relaxant”
- Detuned TENS or “Cefaly” (which is a brand name for an external trigeminal nerve stimulator) [5]

Alternatively, the authors could consider comparing mobilisation of the neck to SMT as separate cohorts, and not in the current method as a pragmatic study, where participants may receive either or both (dependent on practitioner discretion).

Secondly, I believe the study contains significant attention bias difference due to participants receiving five SMT sessions in comparison to no treatment sessions for care as usual (CAU) cohort. This will also potentially add to poor method scores in patient “blinding”, as it should be obvious participants in the SMT cohort, that they have received therapeutic treatment, versus no treatment in the CAU cohort [2].

In addition, there are some concerns over the total number of outcome measures (ten) and poor compliance from participants due to the time to complete all outcome measures thoroughly. I wish the authors good luck in completion of this study.
