# Supplementary material for: Letter to the editor, Odell 2019
Source: Chiropr Man Therap. 2019 Aug 20;27:52. doi: 10.1186/s12998-019-0271-x (PMC6700790; doi:10.1186/s12998-019-0271-x)
Supplement: Related texts

# Reply: Response from the authors

**Authors:** Jim Odell, Carol Clark, Adrian Hunnisett, Osman Hassan Ahmed, Jonathan Branney

The authors would like to thank Associate Professor Tuchin for taking the time to write to the editor about our protocol [1]. The issues raised are important and ones that we considered before undertaking the study.

With regard to the question on placebo arm, we accept that it may have been useful, although on the other hand introducing a valid placebo in any manual therapy randomised controlled trial (RCT) is difficult and rarely removes or accounts for all components of the placebo effect. However, we recognise some studies have attempted to do so [6, 7] and it is a future area for development.

In this study our objective was a pragmatic one, designed to aid real-world clinical decision making on treatment rather than understanding the causal nature of the relationship [8]. The design was therefore ‘add-on’. Some authors have suggested that the aim should be to identify the benefit to the patient regardless of the source; psychosocial or biological, particularly where risk to the patients is low [9, 10].

With regard to the suggestion of comparing mobilisation and spinal manipulative therapy (SMT) this is certainly a study that can be carried out in the future. We chose to explore the effectiveness of manual therapy (MT) (without specifying one type), as it was felt that this is what best mirrors clinical practice, and as such makes this a pragmatic trial.

We are unaware of the use of the specific term ‘attention bias’ but understand the point made. All participants were contacted every week for diary data, often with the non MT group more forthcoming, which may mitigate some of the suggested bias differential. However we are unaware of measures that quantify the impact of ‘attention’ bias. The ‘blinding’ however is an issue with this type of study and will be reflected in discussion of the methodological limitations rather than a score, which is not applicable to the CONSORT process [11, 12].

The potential issues with compliance and outcome measures were considered and efforts made to mitigate these by involving people with chronic migraine in the design of the outcome measure booklets and in trialling the allocation of time to complete. We are able to report that our initial results indicate that the primary outcome measurement instruments were completed thoroughly. However, we noted that not all the migraine diaries were completed. This has provided us with valuable insights for further trials in this population.
